# Supplementary material for: Intracellular Metabolomics Identifies Efflux Transporter Inhibitors in a Routine Caco-2 Cell Permeability Assay—Biological Implications
Source: Cells. 2022 Oct 19;11(20):3286. doi: 10.3390/cells11203286 (PMC9601193; doi:10.3390/cells11203286)
Supplement: Supplementary file 1 [file cells-11-03286-s001.zip › cells-1947958-supplementary.pdf]

Supplementary material

Intracellular metabolomics identifies Efflux transporter inhibitors in a routine Caco-2 cell permeability assay- Biological implications.

Afia Naseem[1], Akos Pal[1], Sharon Gowan[1], Yasmin Asad[1], Adam Donovan[1], Csilla Temesszentandrási-Ambrus[2], Emese Kis[2] Zsuzsanna Gaborik[2], Gurdip Bhalay[1], Florence Raynaud[1,*]

A

% relative transport of Pgp, BCRP and MRP2 probe substrates with Pgp, BCRP and MRP2 inhibitors

		Pgp substrate	BCRP substrate	MRP2 substrate
Main inhibitors	Zosuquidar (5 µM)	**2**	64	66
	Ko143 (10 µM)	57	**2**	62
	MK571 (200 µM)	3	1	**10**
Pgp inhibitors	Valspodar (50 nM)	**19**	120	261
	Ritonavir (10 µM)	**27**	26	233
BCRP inhibitors	FMC (5 µM)	45	**2**	221
	Novobiocin (30 µM)	62	**3**	90
MRP2 inhibitor	Benzbromarone (66.6 µM)	40	4	**10**

B

% relative transport of Pgp, BCRP and MRP2 probe substrates with Pgp, BCRP and MRP2 inhibitors

		Pgp substrate	BCRP substrate	MRP2 substrate
Pgp inhibitors	Elacridar (10 µM)	**8**		
	Chlorpromazine (10 µM)	**74**		
	omeprazole (40 µM)	**69**		
BCRP inhibitors	Febuxostat (10 µM)		**3**	
	atorvastatin (10 µM)		**18**	
	Quercetin (10 µM)		**3**	
MRP2 inhibitors	Rifampicin (200 µM)			**14**
	Quercetin (200 µM)			**52**

Supplementary Table S1: Functional assessment of Pgp, BCRP and MRP2 inhibitors that were used in our experiments to generate the signature. In these experiments, the efflux ratio of Pgp, BCRP and MRP2 substrates was calculated with and without the inhibitors. % relative transport for each inhibitor was then determined with the calculation below:

$$\% \text{ relative transport} = \frac{\textbf{substrate efflux ratio}}{\textbf{substrate+inhibitor efflux ratio}} \times 100\%$$

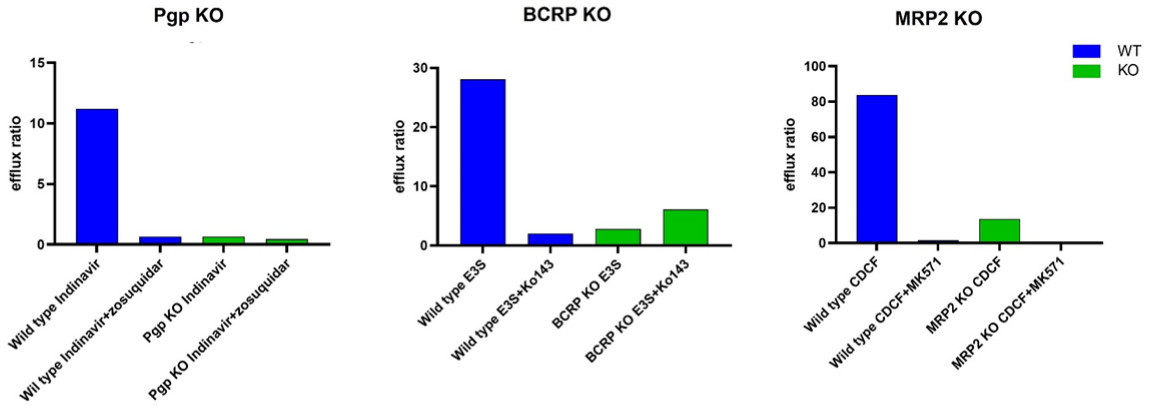

Supplementary Figure S1 Functional assays to evaluate inhibition of transporters in KO cells. In these experiments, the efflux ratio of Pgp, BCRP and MRP2 substrates was calculated in the KO /WT cells (C2BBe1) obtained from SOLVO Biotechnology. The data shows that the efflux ratio of probe substrates was markedly reduced in KO cells

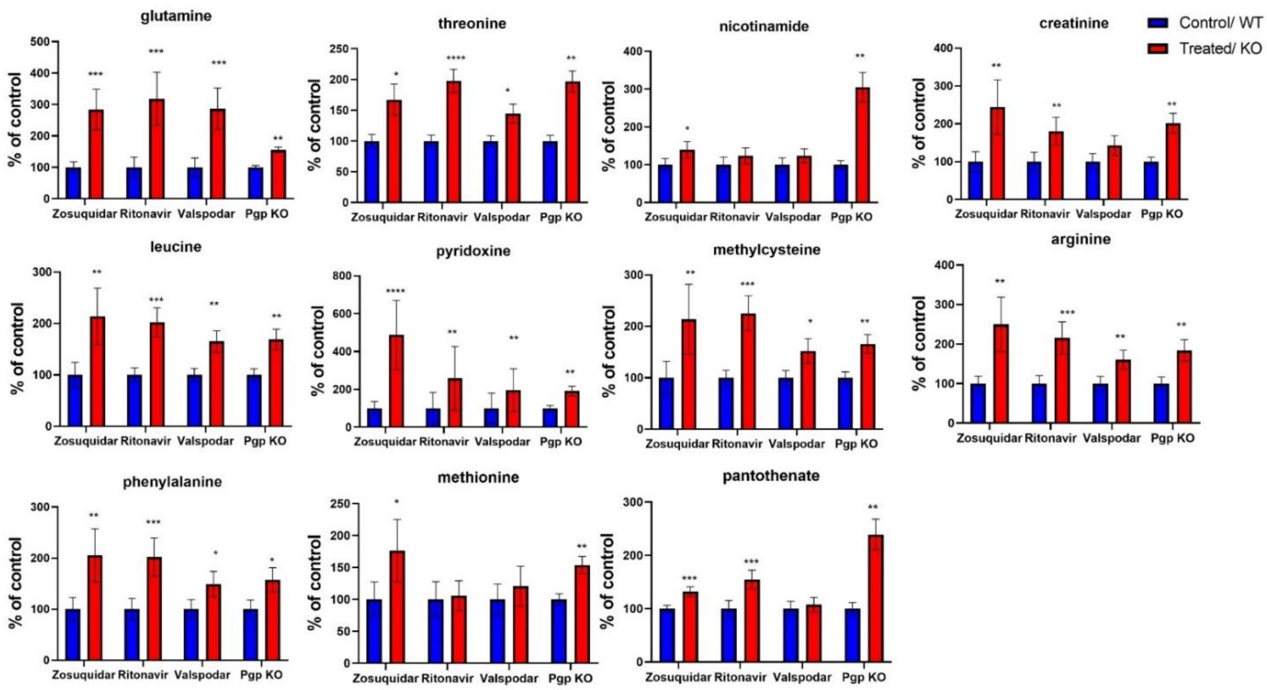

Supplementary figure S2 representing changes in metabolites induced by Pgp inhibition in cells. The data represent changes in metabolites in response to Pgp KO out and Pgp inhibition by zosuquidar (5 µM), ritonavir (10 µM), valspodar (50 nM). The data representing changes with zosuquidar inhibition was acquired from 3 individual experiments, each experiment was performed with 6 minimum replicates and the data is presented as mean of 3 repeats. The data from other inhibitors and Pgp KO represents mean of 1 independent experiment which was performed with at least 6 replicates. The data was normalised by dividing the mean (peak area) of treated /KO group with the control group and is presented as a percentage (mean ±SEM) of the control group. Significance of these results was determined using Mann-Whitney test and results with *p<0.05, **p<0.01, ***p<0.001, ****p<0.0001 were considered significant.

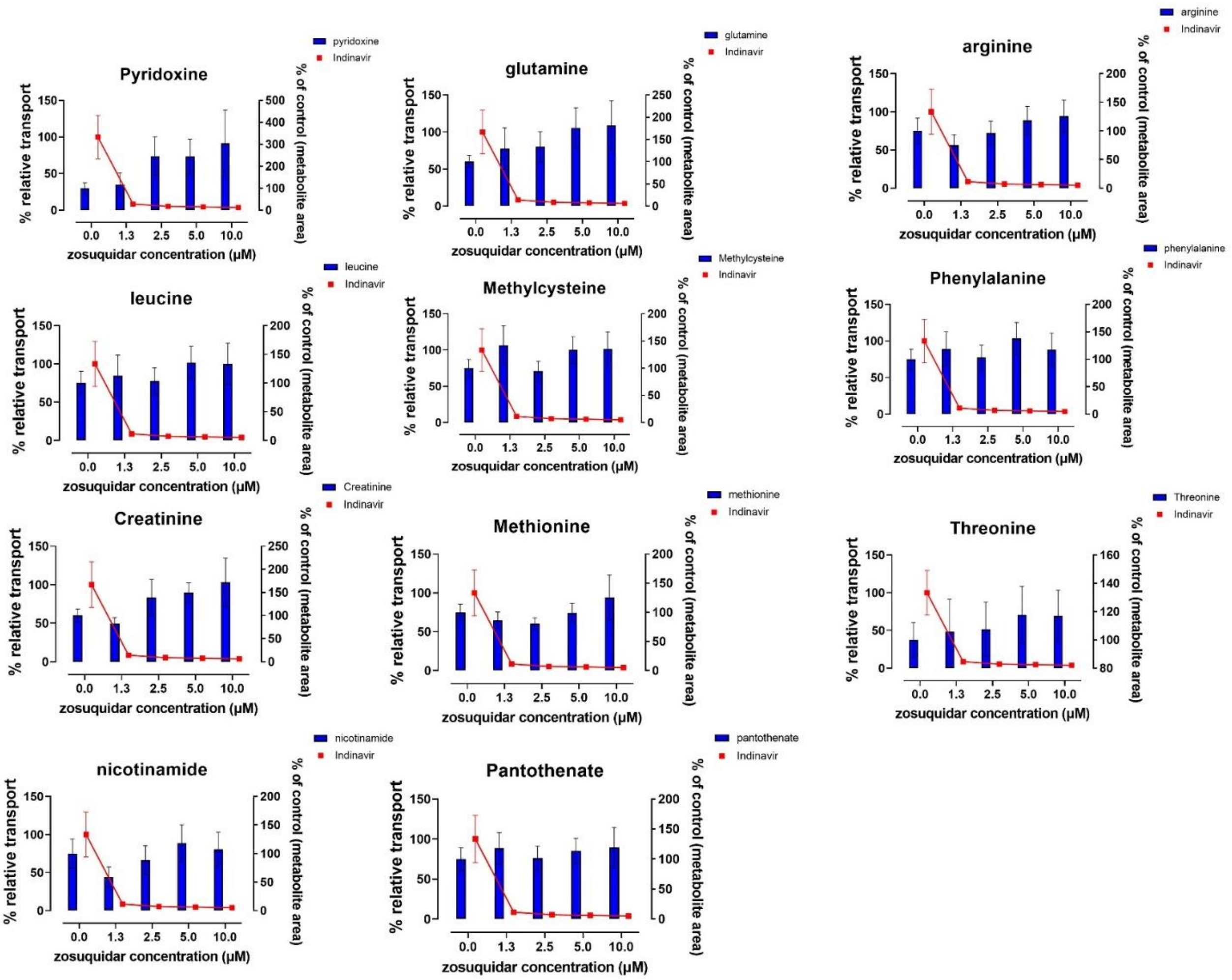

Supplementary figure S3 representing changes in Pgp signature metabolites and Indinavir in Caco-2 cells treated with increasing concentration of zosuquidar. The data was acquired from 3 individual experiments, each experiment was performed with 3 minimum replicates and the data is presented as mean of 3 repeats. The metabolomics data was normalised by dividing the mean (peak area) of treated group with the control group and is presented as a percentage (mean ±SEM) of the control group.

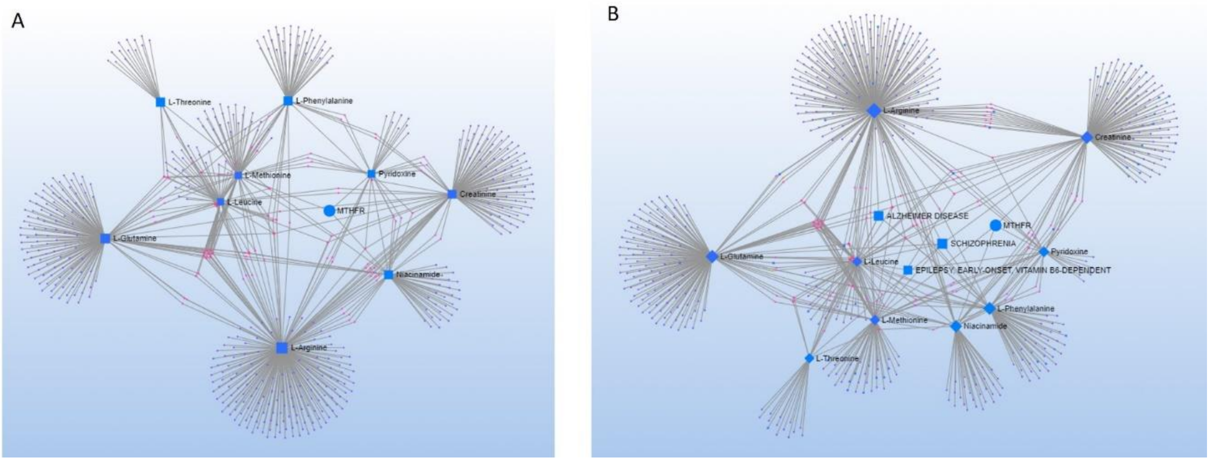

Supplementary Figure S4 network analysis using Pgp signature metabolites(A) gene-metabolite network analysis based on Pgp signature metabolites **(B)** metabolite-disease analysis based on Pgp signature metabolites indicating a link between Pgp signature metabolites and neurological diseases

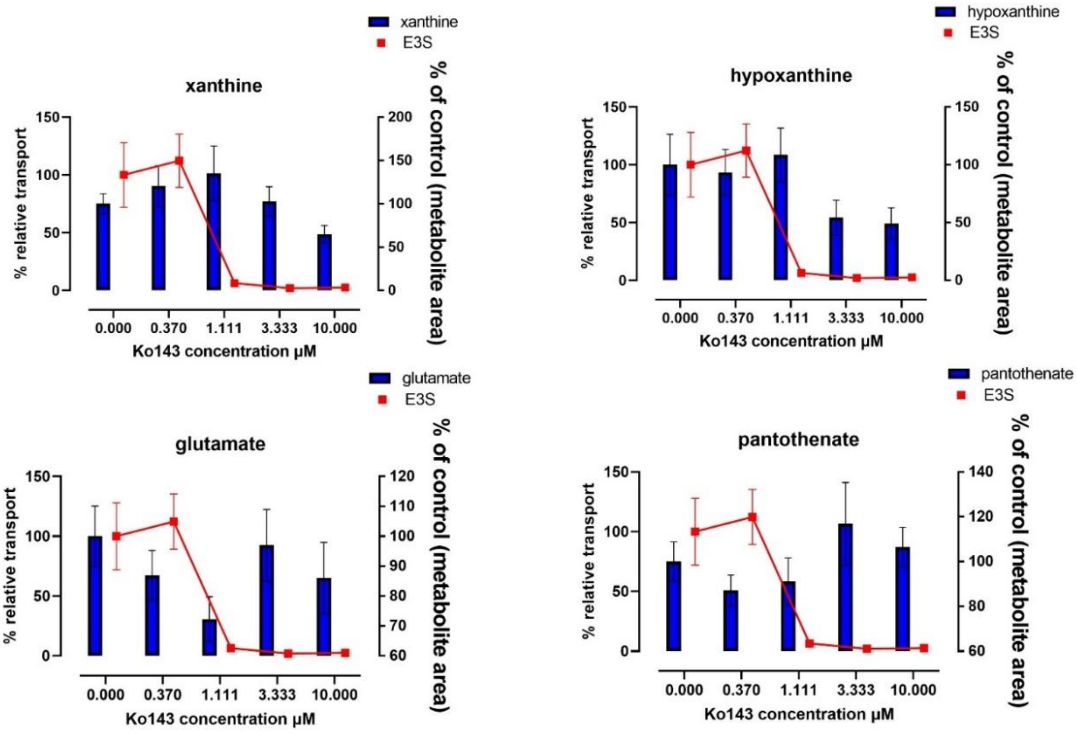

Supplementary figure S5 representing changes in BCRP signature metabolites and E3S in Caco-2 cells treated with increasing concentration of the Ko143. The data was acquired from 1 experiment performed with 6 minimum replicates. The metabolomics data was normalised by dividing the mean (peak area) of treated group with the control group and is presented as a percentage (mean ±SEM) of the control group. The functional data was acquired from 3 individual experiments, each experiment was performed with 3 replicates and the data is presented as mean ±SEM of 3 repeats.

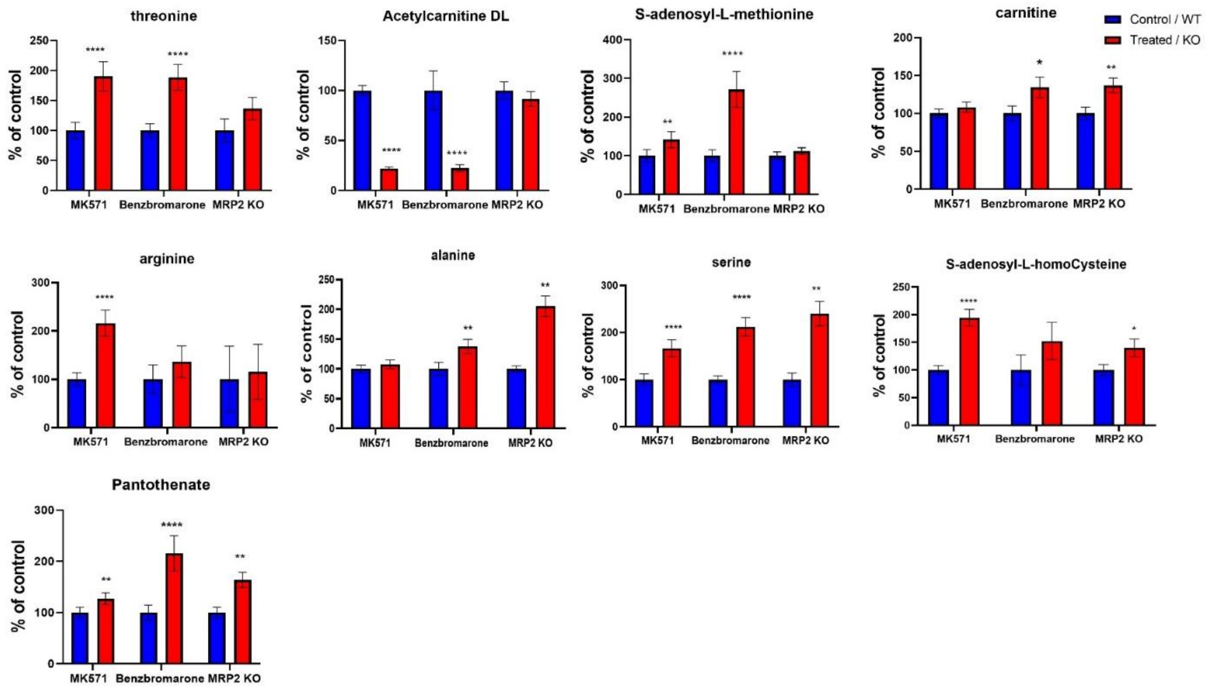

Supplementary figure S6 representing changes in metabolites in cells treated with MRP2 inhibitors and MRP2 KO cells. The data represent changes in metabolites in response to MRP2 KO and MRP2 inhibition by MK571 (200 µM) and benzbromarone (66.6 µM). The data representing changes with MK71 inhibition was acquired from 3 individual experiments, each experiment was performed with 6 minimum replicates and the data is presented as mean of 3 repeats. The data from benzbromarone and MRP2 KO represents mean of 1 independent experiment which was performed with at least 6 replicates. The data was normalised by dividing the mean (peak area) of treated /KO group with the control group and is presented as a percentage (mean ±SEM) of the control group. Significance of these results was determined using Mann-Whitney test and results with *$p<0.05$, **$p<0.01$, ***$p<0.001$, ****$p<0.0001$ were considered significant

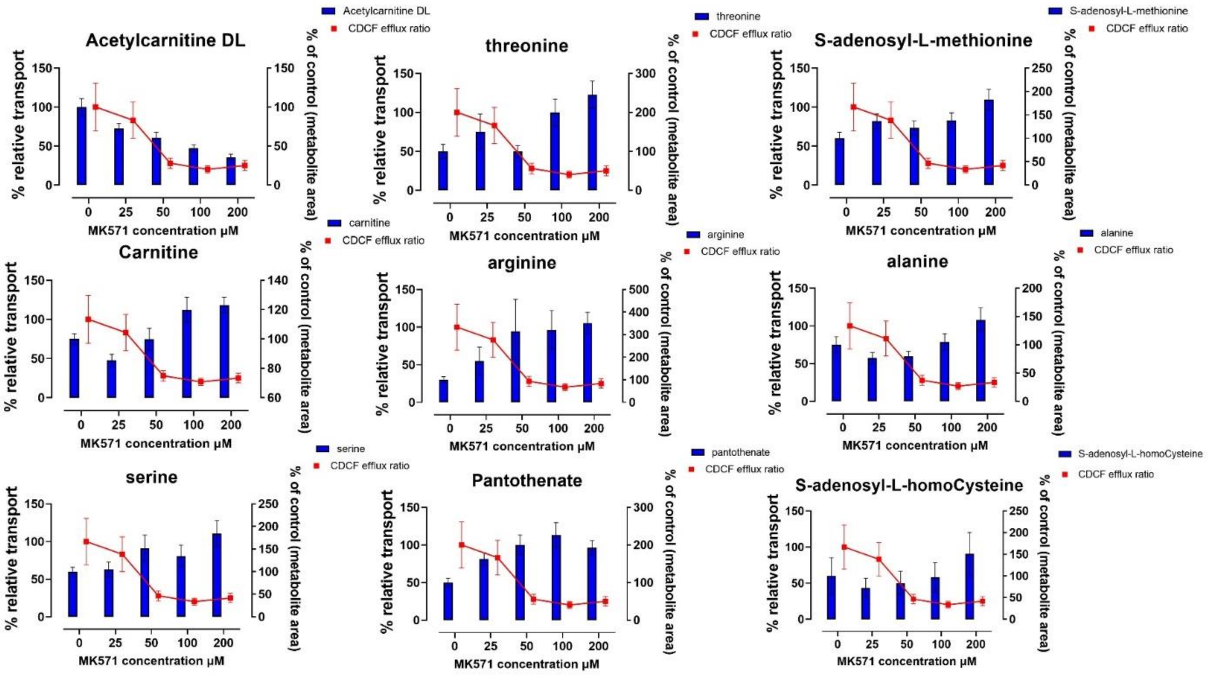

Supplementary figure S7 representing changes in MRP2 signature metabolites and CDCF in Caco-2 cells treated with increasing concentration of MK571. The data was acquired from 1 experiment performed with 6 minimum replicates. The metabolomics data was normalised by dividing the mean (peak area) of treated group with the control group and is presented as a percentage (mean ±SEM) of the control group. The functional data was acquired from 3 individual experiments, each experiment was performed with 3 replicates and the data is presented as mean ±SEM of 3 repeats.

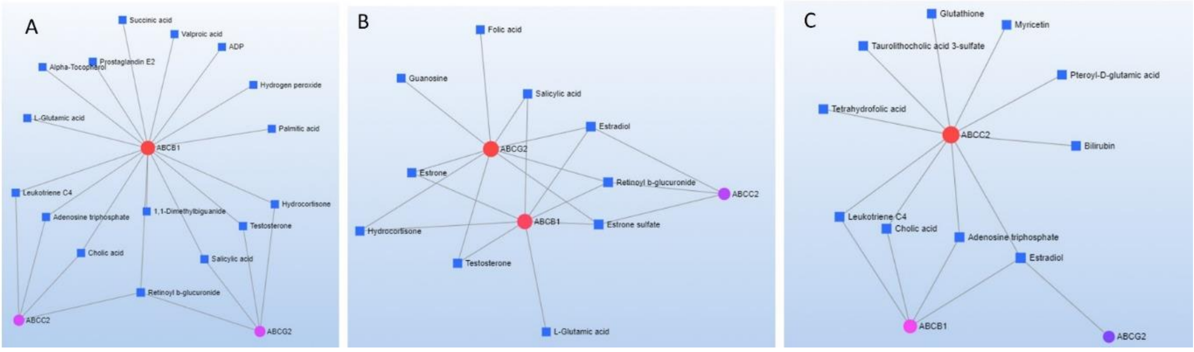

Supplementary Figure S8: Data derived from publicly available database used in signature network analysis: **(A)** metabolites associated with Pgp transporter from public database **(B)** metabolites associated with BCRP transporter from public database **(C)** metabolites linked to MRP2 transporter

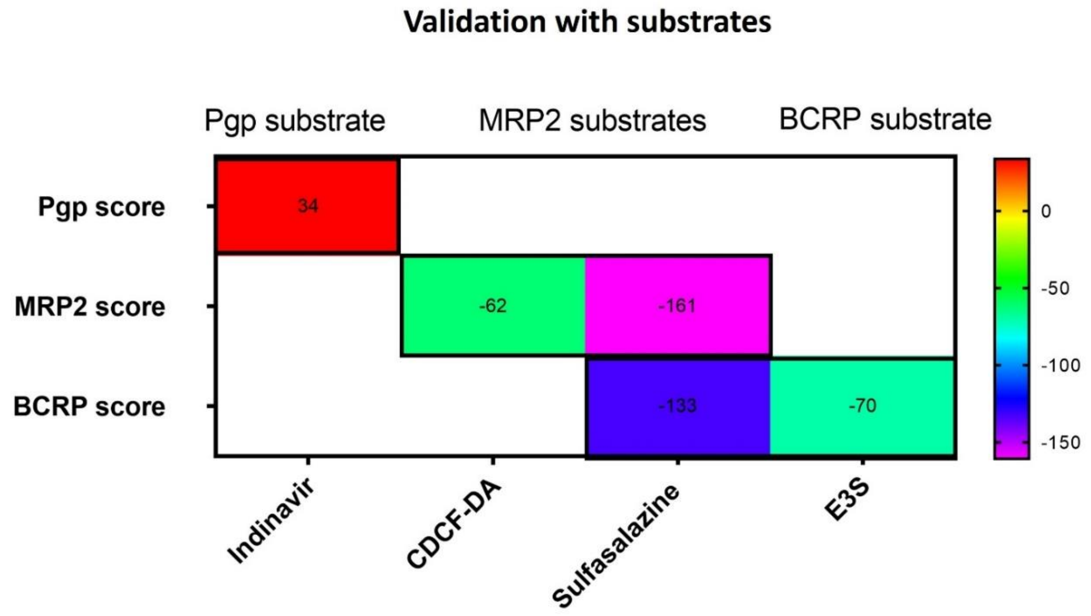

Supplementary Figure S9: Validation of scoring system with Pgp, BCRP and MRP2 substrates. The data was acquired from 1 biological experiment with minimum 6 replicates.

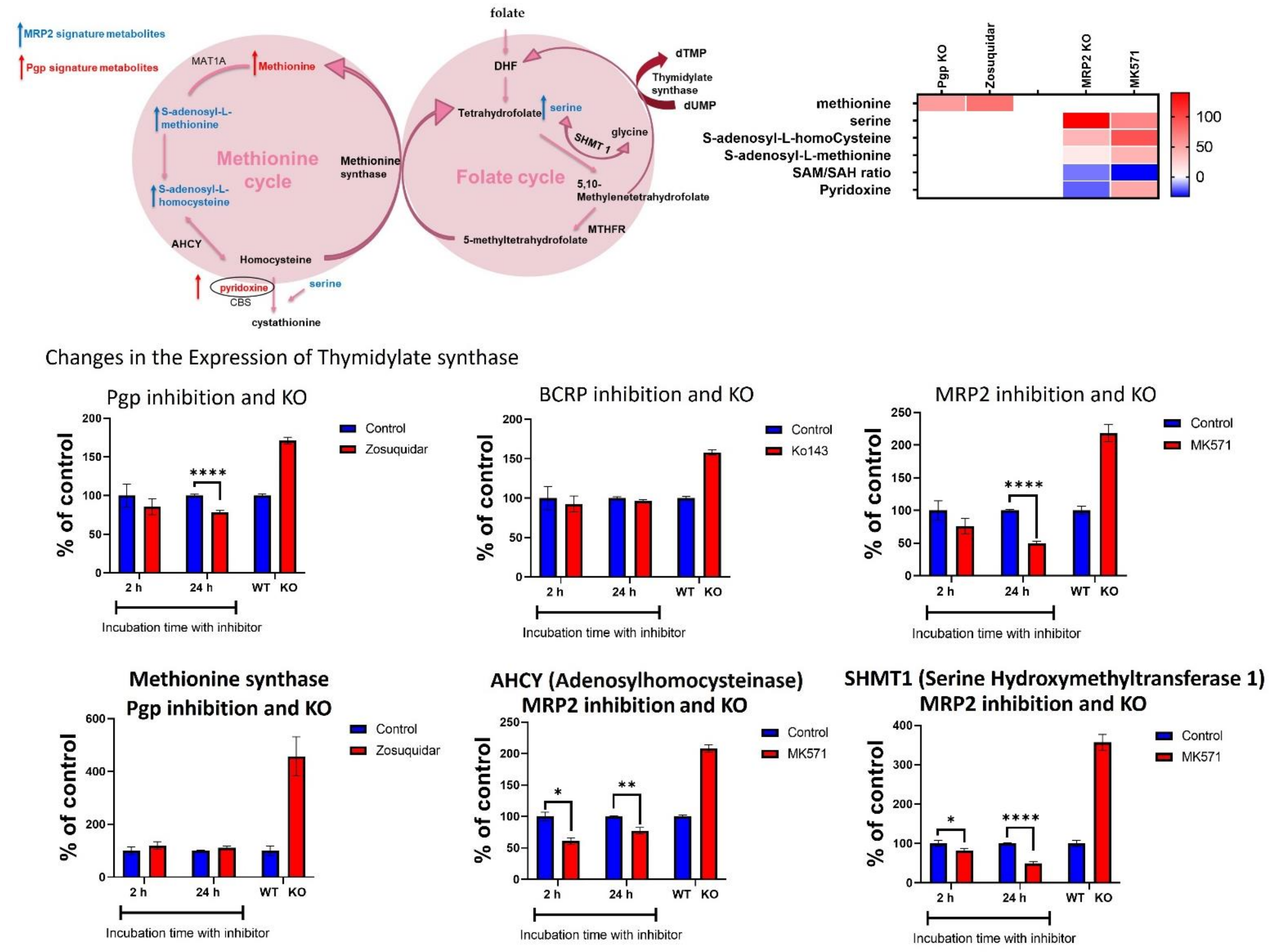

Supplementary Figure S10: Thymidylate synthase, SHMT1, methionine synthase and AHCY expression in cells treated with zosuquidar (5 µM), MK571 (200 µM) and Ko143 (10 µM) over 2 and 24 h and in cells with Pgp, BCRP KO and MRP2 KO. Cells were grown in a transwell plate for 2 h incubation and in 6 well plate for 24 h incubation. Cells were incubated with Zosuquidar, MK571 and Ko143 for 2 h and 24 h after which cells were lysed and protein was extracted 1.2 µg protein was loaded on WES/JESS instrument and β Actin or vinculin was used as a loading control. The data is normalized against β Actin or vinculin and is represented an average of minimum 3 replicates from one experiment with KO cells and 2 h inhibition with inhibitors. The data representing changes with 24 h incubation with inhibitors is an average of 2 biological repeats. Significance of these results was determined using Mann-Whitney test and results with *p<0.05, **p<0.01, ***p<0.001, ****p<0.0001 were considered significant.
